# Development and validation of a hypoxia-immune-based microenvironment gene signature for risk stratification in gastric cancer

**DOI:** 10.1186/s12967-020-02366-0

**Published:** 2020-05-14

**Authors:** Yifan Liu, Jianhua Wu, Weiwei Huang, Shaowen Weng, Baochun Wang, Yiming Chen, Hao Wang

**Affiliations:** 1 The First Department of Gastrointestinal Surgery, Hainan General Hospital, Hainan Affiliated Hospital of Hainan Medical University, Haikou, China; 2 Department of General Surgery, Shanghai Ninth People’s Hospital, School of Medicine, Shanghai Jiao Tong University, Shanghai, China; 3 Department of Oncology, Nanfang Hospital, Southern Medical University, Guangzhou, China

**Keywords:** Gastric cancer, Microenvironment, Hypoxia, Immune, Prognosis, Prediction

## Abstract

**Background:**

Increasing evidences have found that the clinical importance of the interaction between hypoxia and immune status in gastric cancer microenvironment. However, reliable prognostic signatures based on combination of hypoxia and immune status have not been well-established. This study aimed to develop a hypoxia-immune-based gene signature for risk stratification in gastric cancer.

**Methods:**

Hypoxia and immune status was estimated with transcriptomic profiles for a discovery cohort from GEO database using the *t*-SNE and ESTIMATE algorithms, respectively. The Cox regression model with the LASSO method was applied to identify prognostic genes and to develop a hypoxia-immune-based gene signature. The TCGA cohort and two independent cohorts from GEO database were used for external validation.

**Results:**

Low hypoxia status (*p* < 0.001) and high immune status (*p* = 0.005) were identified as favorable factors for patients’ overall survival. By using the LASSO model, four genes, including CXCR6, PPP1R14A and TAGLN, were identified to construct a gene signature for risk stratification. In the discovery cohort (n = 357), patients with low risk yielded better outcomes than those with high risk regarding overall survival across and within TNM stage subgroups. Multivariate analysis identified the hypoxia-immune-based gene signature as an independent prognostic factor (*p* < 0.001). A nomogram integrating the gene signature and known risk factors yielded better performance and net benefits in calibration and decision curve analyses. Similar results were validated in the TCGA (n = 321) and two independent GEO (n = 300 and n = 136, respectively) cohorts.

**Conclusions:**

The hypoxia-immune-based gene signature represents a promising tool for risk stratification tool in gastric cancer. It might serve as a prognostic classifier for clinical decision-making regarding individualized prognostication and treatment, and follow-up scheduling.

## Background

Gastric cancer is a common cancer and a leading cause of cancer-related deaths worldwide [1]. The clinicopathologic characteristics are routinely revealed though Lauren/WHO classification and tumor-node-metastasis (TNM) staging system for prognostication which is also critical for the selection of appropriate treatment [2]. However, gastric cancer is a heterogeneous disease, and its outcomes can vary significantly even for patients with similar clinical features and treatment regimens, suggesting that clinicopathologic characteristics and current classifications are insufficient for prognostication and risk stratification [3, 4]. Hence, identification of novel markers providing more predictive value is highly demanded for improving the prognostication for gastric cancer.

Gastric cancer tissue is highly heterogeneous, where malignant cells are in an intricate relationship with tumor microenvironment, including immune cells, vessels and fibroblasts [5–7]. Either through structural and functional abnormality of tumor vasculature or deterioration of the diffusion geometry of blood vessels, the vessels and fibroblasts cells of tumor microenvironment influence O_2_ perfusion and diffusion, and therefore, leading to the development of hypoxia in that tissue area [8]. Hypoxia has been reported as one of characteristic hallmarks of solid tumors that directly contribute to the malignant properties of cancers, including tumor progression, invasion and metastasis [9–12]. Meanwhile, immune cell is also a potentially powerful force that can prevent or slow tumor growth, which is associated with tumor invasion and metastasis [13–15]. Interestingly, increasing evidences have found that the direct or indirect interaction between hypoxia and immune status in gastric cancer microenvironment [16, 17], although their underlying mechanisms remains unclear.

In this study, we speculated that immune and hypoxia interaction could provide prognostic value for gastric cancer patients. Through a series of systematic analyses, we developed a novel gene signature by incorporating immune and hypoxia status into the current clinicopathologic characteristics and staging system, aimed to improve the prognostication of gastric cancer.

## Methods

### Patient cohort and data preparation

The discovery cohort contained 357 gastric cancer patients retrieved from the Gene Expression Omnibus (GEO, available at: https://www.ncbi.nlm.nih.gov/geo/) database (GSE84437). Three independent cohorts were used for external validations. The Cancer Genome Atlas (TCGA) cohort contained 321 patients from the “TCGA-STAD” project and the corresponding level-3 gene expression data were obtained from the Genomic Data Commons (available at: https://portal.gdc.cancer.gov) Data Portal on Nov 11, 2019. The ACRG cohort included 300 patients from the Asian Cancer Research Group study (GSE66229). To examine the survival benefit of chemotherapy for patients in different risk groups, a cohort containing 136 patients (named as “CHEM” cohort) from the GEO database (GSE15459) was used for further analyses. The study complied with the principles set forth in the Declaration of Helsinki. Access to the de-identified linked dataset was obtained from the TCGA and GEO databases in accordance with the database policy. For analyses of de-identified data from the TCGA and GEO databases, institutional review board approval and informed consent were not required.

For all expression datasets from the GEO database, background correction and quartile normalization were performed for each series by applying the robust multi-array average algorithm [18]. The average value of gene symbols with multiple probes was calculated as expression level. For datasets from TCGA database, mRNA expression was quantified with fragments per kilobase of exon per million reads mapped (FPKM). For all cohorts, only patients with available expression profiles, clinicopathologic and survival data were included for analyses. The primary prognosis endpoint was overall survival and survival curves were estimated using the Kaplan–Meier method.

### Identification of hypoxia status and hypoxia-related DEGs

To deduce the hypoxia status, an algorithm of *t*-distributed Stochastic Neighbor Embedding (*t*-SNE) was applied [19]. *t*-SNE, a nonparametric, unsupervised method, can divide or condense patients into several distinct clusters, based on given signatures or hallmarks. The hallmark gene sets of hypoxia including 200 genes, were downloaded from the Molecular Signatures Database (MSigDB version 6.0). Based on the clusters, two groups including “hypoxia^high^” and “hypoxia^low^” groups were identified to estimate the hypoxia status. Further, expression changes of target genes involved in HIF-1 signaling pathway were analyzed between the hypoxia^high^ and hypoxia^low^ groups to explore their association with hypoxia. These targets were retrieved from the Kyoto Encyclopedia of Genes and Genomes (KEGG) database (https://www.kegg.jp/; ID:04066), including 15 genes involved in “Increase oxygen delivery” and 11 genes related to “Reduce oxygen consumption”. The limma algorithm was used to identify differentially expressed genes (DEGs) between the two groups [20]. Genes with a false discovery rate (FDR) adjusted *p*-value < 0.0001 and an absolute value of log2 (fold change) > 1 were considered as hypoxia-related DEGs.

### Identification of immune status and immune-related DEGs

The newly developed algorithm, ESTIMATE (Estimation of STromal and Immune cells in MAlignant Tumor tissues using Expression data), takes advantages of characteristics of the transcriptomic profiles of tumor tissues to infer the proportion of different infiltrating stromal and immune cells [21]. In this study, the ESTIMATE method was applied to impute an immune score to represent the infiltration of immune cells for each gastric cancer sample to predict the immune status. Based on the immune scores, patients were stratified into two groups, and their prognoses were examined and compared. To identify the optimal score cutoff for dividing patients with the most significantly different outcomes, a method of maximally selected rank statistics was employed by using an R package “maxstat” [22]. Based on the optimal cutoff, patients with high immune scores were classified into the “immune^high^” group, and those yield lower immune scores were considered as “immune^low^” group. DEGs between the immune^high^ and immune^low^ groups were identified by the limma method. Genes with an FDR-adjusted *p*-value < 0.0001 and an absolute value of log2 (fold change) > 1 were considered as immune-related DEGs.

### Identification of hypoxia-immune-related prognostic DEGs

The hypoxia and immune status identified above was further combined into a two-dimension index, whereby patients were divided into three groups, i.e., hypoxia^low^/immune^high^, hypoxia^high^/immune^low^, and “mix” groups. The hypoxia-immune-related DEGs were obtained by the expression comparison between the hypoxia^low^/immune^high^ and hypoxia^high^/immune^low^ groups (|log2FC| > 1.7, FDR-adjusted *p* < 0.0001). Two gene sets (i.e., protective and risk DEGs) were then developed by overlapping the hypoxia-immune-related DEGs and immune/hypoxia-related DEGs obtained above. The protective DEGs contained all DEGs highly expressed in hypoxia^low^/immune^high^ group and also with conserved overexpression in hypoxia^low^ or immune^high^ group. And those DEGs overexpressed in hypoxia^high^/immune^low^ group as well as in hypoxia^high^ or immune^low^ group were considered as risk DEGs. To obtain hypoxia-immune-related prognostic DEGs, univariate Cox regression analyses were further performed among all protective and risk DEGs. Those with a *p* < 0.0001 and hazard ratio < 0.2 (for protective DEGs) or > 2 (for risk DEGs) were considered as significant.

### Derivation of hypoxia-immune-based gene signature and prognosis classifier

The Least Absolute Shrinkage and Selection Operator (LASSO) is a popular method for regression with high-dimensional predictors, which can preserve valuable variables and avoid overfitting. This approach has been extended and broadly applied to the Cox proportional hazard regression model for survival analysis with high-dimensional data [23, 24]. In this study, the LASSO Cox regression model was employed to select the most prognostic gene signature from all the identified hypoxia-immune-related prognostic DEGs within the discovery cohort. Three-fold cross-validation and 1000 iterations were conducted to reduce the potential instability of the results. The optimal tuning parameter λ was identified via 1-SE (standard error) criterion. Then, a prognosis classifier was developed based on the individual-level risk scores derived from the selected prognostic gene signature. For each patient, the risk score was a sum of the products of the expression levels of the prognostic signature genes and the corresponding coefficients derived from LASSO model, i.e., risk score = ∑(coefficient_i_ × expression of signature gene_i_). Based on the individual-level risk scores, an optimal cutoff was identified via the method of maximally selected rank statistics to develop a prognosis classifier for gastric cancer patients.

### Statistical analysis

All analyses were performed with R version 3.4.1 (http://www.R-project.org) and its appropriate packages. *t*-SNE algorithm was performed by using R package “Rtsne” based on nonlinear dimensionality reduction. Immune score was imputed by using the “estimate” package. The Lasso Cox regression modeling was conducted by using the “glmnet” package. Data were analyzed with standard statistical tests as appropriate. Multiple testing was adjusted by the FDR method. For identifying independent risk factors for survival, multivariate Cox regression analysis was performed to adjust covariates.

## Results

### Hypoxia status and hypoxia-related DEGs in gastric cancer

**Fig. 1:**
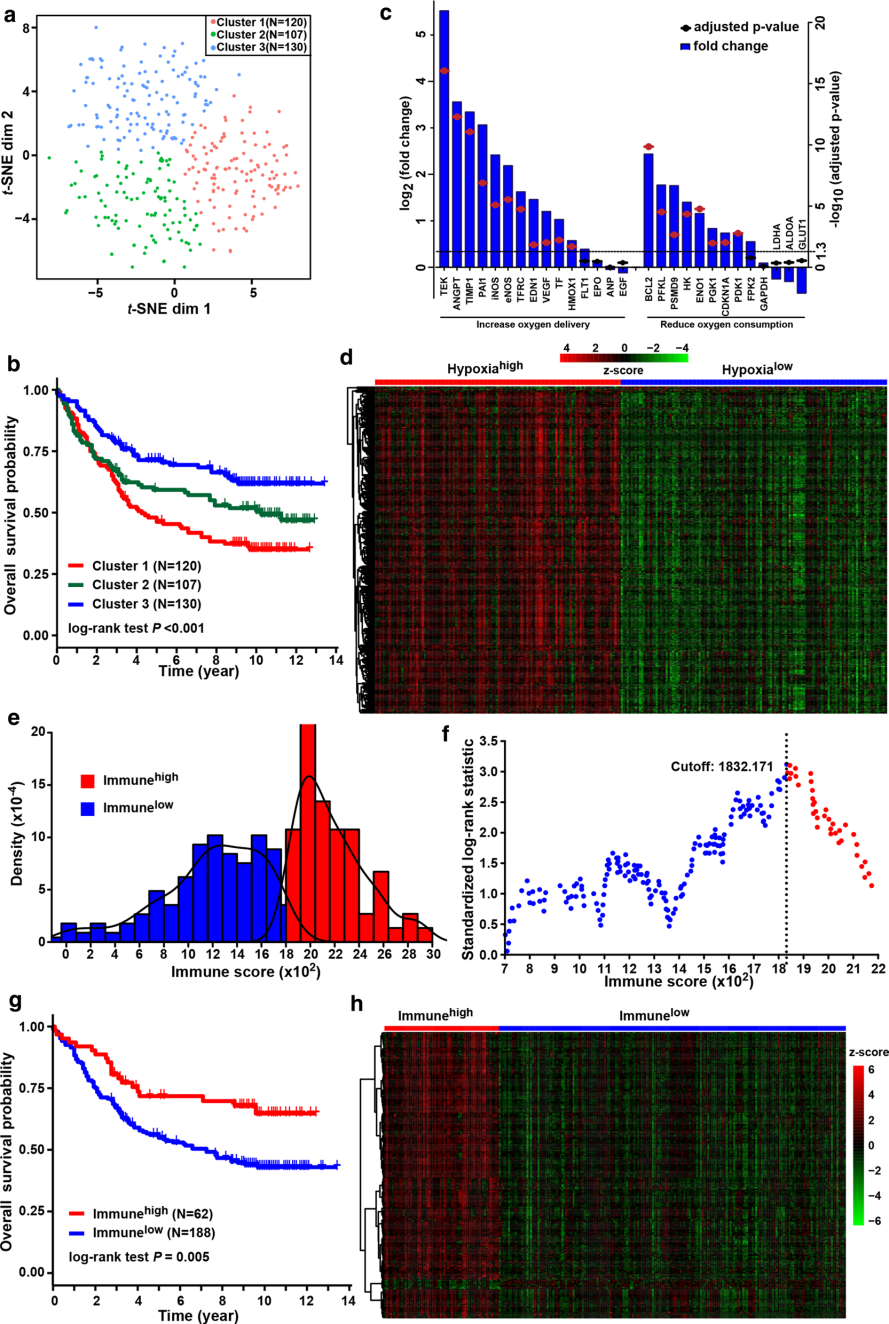
Identification of hypoxia and immune status and hypoxia- and immune-related DEGs. **a** Dot plot for three distinct clusters identified by *t*-SNE algorithm based on 200 hypoxia hallmark genes. **b** Kaplan–Meier plot of overall survival for patients in three clusters. **c** Expression changes (hypoxia^high^ vs. hypoxia^low^) of target genes involved in HIF-1 KEGG pathway. **d** Heatmap showing expression profiles for hypoxia-related DEGs with comparison between hypoxia^high^ and hypoxia^low^ groups. **e** Histogram shows the density distribution for high- and low-immune score groups divided by the optimal cutoff. **f** Scatter plot shows the standardized log-rank statistic value for each corresponding immune score cutoff. The optimal cutoff with the maximum standard log-rank statistic is marked with a vertical dashed line. **g** Kaplan–Meier plot of overall survival for patients in immune^high^ and immune^low^ groups. **h** Heatmap showing expression profiles for immune-related DEGs with comparison between immune^high^ and immune^low^ groups

The discovery cohort contained 357 gastric cancer patients from the GEO database. Patient clinicopathologic characteristics are listed in Table 1. The tumor stage upon presentation was stage I in 5.9%, stage II in 30.5%, and stage III in 63.6% of cases. With the expression matrix of 200 hypoxia hallmark genes from the MSigDB version 6.0, the Euclidean distance was calculated between any two patients in the discovery cohort and condensed into two-dimensional points using a nonlinear dimensionality reduction algorithm *t*-SNE (see Methods for details); and subsequently, three patient clusters were determined and each patient was assigned to its closest (Fig. 1a). There were 120,107 and 130 patients in the three distinct clusters (i.e., Cluster1, Cluster2 and Cluster3), respectively. Survival comparison showed significantly differences among the three clusters (log-rank test, *p* < 0.001). Patients in Cluster3 yield the best overall survival while those in Cluster1 had the worst prognosis outcome (Fig. 1b). This indicated that Cluster3 and Cluster1 might be in the lowest and highest status of hypoxia. We further explore the expression changes (hypoxia^high^ vs. hypoxia^low^) of the target genes from the KEGG HIF-1 signaling pathway. Two gene sets were used, included genes involved in “Increase oxygen delivery” (15 genes) and “Reduce oxygen consumption” (13 genes). Among 15 genes related to “Increase oxygen delivery”, 11 (73.33%) were overexpressed in the hypoxia^high^ group, compared against with the hypoxia^low^ group. And 8 of 13 (61.54%) genes related to “Reduce oxygen consumption” were overexpressed in the hypoxia^high^ group (Fig. 1c). These results showed the defined groups were significantly associated with the hypoxia status. Accordingly, patients in Cluster1 and Cluster3 were classified into “hypoxia^high^” and “hypoxia^low^” groups, respectively.

**Table 1:** Baseline characteristics of patients in the discovery cohort

Characteristics	Whole cohort (n = 357)	Log-rank *p*	Low risk (n = 95)	High risk (n = 262)	*p*
Gender		0.203			0.427
Male	242 (67.79)		68 (71.58)	174 (66.41)	
Female	115 (32.21)		27 (28.42)	88 (33.59)	
Age		0.051			0.391
< 65 years	229 (64.15)		57 (60.00)	172 (65.65)	
≥ 65 years	128 (35.85)		38 (40.00)	90 (34.35)	
TNM stage		< 0.001			0.004
I	21 (5.88)		10 (10.53)	11 (4.20)	
II	109 (30.53)		37 (38.95)	72 (27.48)	
III	227 (63.59)		48 (50.53)	179 (68.32)	
t-SNE clustering		< 0.001			< 0.001
Cluster1	120 (33.61)		12 (12.63)	108 (41.22)	
Cluster2	107 (29.97)		26 (27.37)	81 (30.92)	
Cluster3	130 (36.41)		57 (60.00)	73 (27.86)	
Hypoxia status^a^		< 0.001			< 0.001
High	120 (33.61)		12 (12.63)	108 (41.22)	
Low	130 (36.41)		57 (60.00)	73 (27.86)	
Immune status^a^		0.005			< 0.001
Low	188 (52.66)		31 (32.63)	157 (59.92)	
High	62 (17.37)		38 (40.00)	24 (9.16)	
Risk group by classifier		< 0.001			–
Low	95 (25.3)		95 (100.00)	0 (0.00)	
High	262 (74.7)		0 (0.00)	262 (100.00)	

^a^Patients (n = 107) in *t*-SNE-derived cluster2 considered as in “moderate hypoxia status” were excluded from the comparison

Expression profiles were compared between the hypoxia^high^ and hypoxia^low^ groups to obtain hypoxia-related DEGs. A total of 372 hypoxia-related DEGs were identified (Fig. 1d). Among them, 368 (98.9%) was overexpressed in the hypoxia^high^ group where patients yielded relatively worse survival. These DEGs were considered as hypoxia-related risk DEGs. And four DEGs were found with overexpression in the hypoxia^low^ group where patients yielded better prognosis. They were regarded as hypoxia-related protective DEGs. Overall, among all the hypoxia-related DEGs, most of them were considered as risk factors.

### Immune status and immune-related DEGs in gastric cancer

Immune status was identified for 250 patients in the hypoxia^high^ and hypoxia^low^ groups, based on the infiltration of immune cells in tumor tissue. Immune scores were calculated to represent the proportion of infiltrating immune cells by the ESTIMATE method (see Methods for details). Among all patients, the estimated immune score ranged from − 145.8 to 2910.8. The optimal cutoff was identified to classify patients into two groups (i.e., immune^high^ and immune^low^ groups) with the most distinct survivals by a method based on maximally selected rank statistics (Fig. 1e, f). Patients in the immune^high^ group yielded better survival than those in the immune^low^ group (log-rank test, *p* = 0.005) (Fig. 1g).

Immune-related DEGs were obtained by expression comparison between the immune^high^ and immune^low^ groups. A total of 216 immune-related DEGs were identified (Fig. 1h). Among them, 209 (96.8%) was overexpressed in the immune^high^ group where patients yielded relatively better survival. These DEGs were considered as immune-related protective DEGs. And seven DEGs were found with overexpression in the immune^low^ group where patients yielded worse prognosis. They were regarded as immune-related risk DEGs. Overall, among all the immune-related DEGs, most of them were considered as protective factors.

### Hypoxia-immune-related prognostic DEGs in gastric cancer

**Fig. 2:**
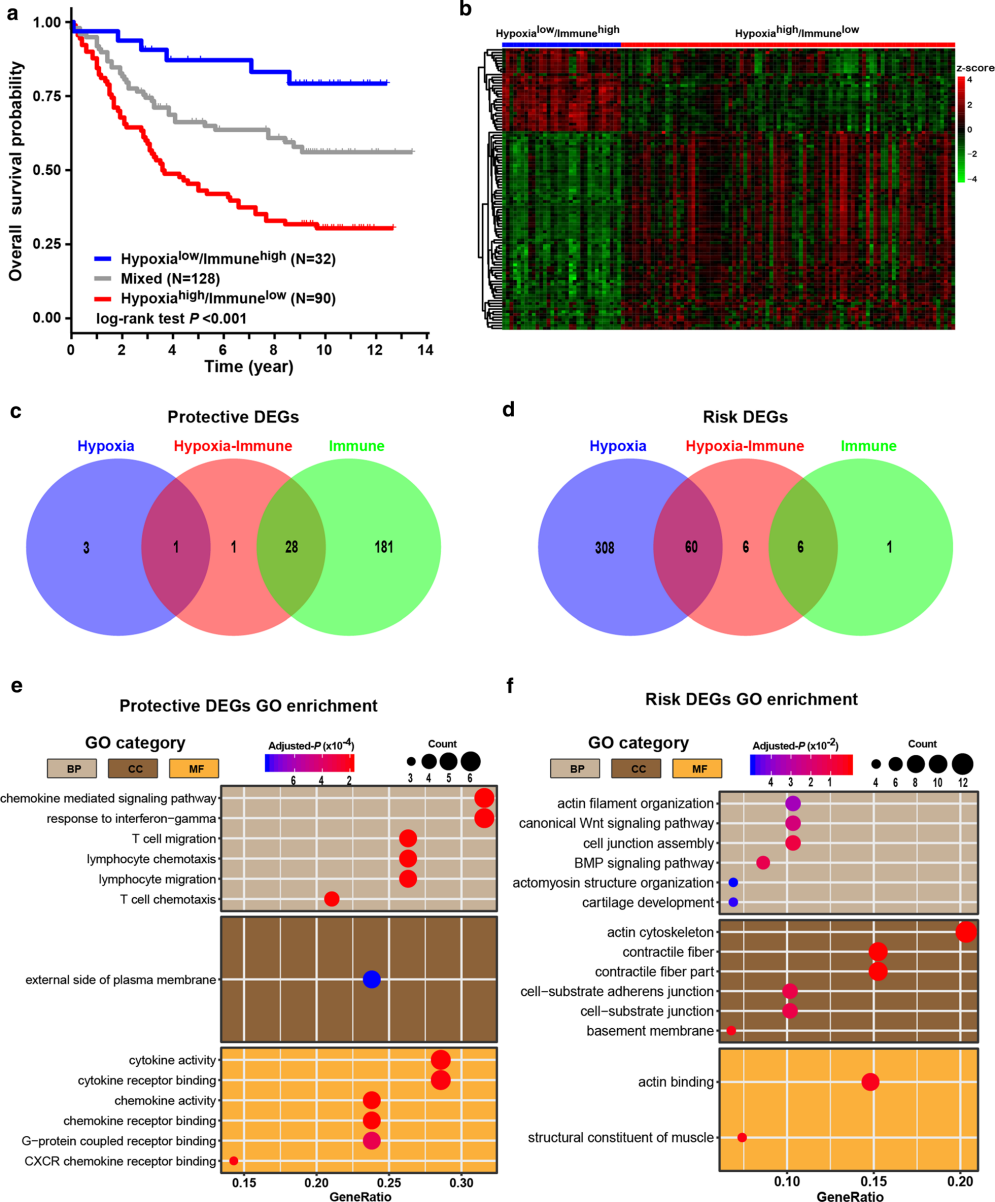
Identification and biological function of hypoxia-immune-related protective and risk DEGs. **a** Kaplan–Meier plot of overall survival for patients in three groups by combining the hypoxia and immune status. **b** Heatmap showing expression profiles for hypoxia-immune-related DEGs with comparison between hypoxia^low^/immune^high^ and hypoxia^high^/immune^low^ groups. **c**, **d** Venn diagrams show overlaps of hypoxia-immune-related DEGs with hypoxia-related and immune-related DEGs for identification of protective and risk DEGs. **e**, **f** Representative Gene Ontology terms enriched by the hypoxia-immune-related protective and risk DEGs. *P*-values were adjusted by false discovery rate

According to the above hypoxia and immune status, we further combined them into a two-dimension index, whereby patients were divided into three groups: hypoxia^low^/immune^high^, hypoxia^high^/immune^low^, and mix groups. Survival analysis showed significant differences among three groups (log-rank test, *p* < 0.001); patients in the hypoxia^low^/immune^high^ group had the best survival while those in the hypoxia^high^/immune^low^ group yield the worst prognosis (Fig. 2a). This provided a hint of inverse association between effects of hypoxia and immune on prognosis of gastric cancer patients.

To obtain hypoxia-immune-related DEGs, expression profile comparison was further conducted between the hypoxia^low^/immune^high^ and hypoxia^high^/immune^low^ groups. A total of 102 hypoxia-immune-related DEGs were identified (Fig. 2b), including 30 overexpressed in hypoxia^low^/immune^high^ groups where patients yielded better survival, which were defined as hypoxia-immune-related protective DEGs, and 72 overexpressed in hypoxia^high^/immune^low^ group where patients had worse outcome, which were defined as hypoxia-immune-related risk DEGs.

Two gene sets (protective DEGs and risk DEGs) were developed by combining the hypoxia-immune-related DEGs and immune- (or hypoxia-) related DEGs obtained above. The conserved DEGs in above two processes of DEGs identification were considered as critical protective or risk DEGs (see Methods for details). Finally, a total of 95 critical DEGs were identified, including 29 protective DEGs (Fig. 2c) and 66 risk DEGs (Fig. 2d). Among those critical protective DEGs, almost all of them (28 out of 29, 96.6%) were immune-related DEGs. On the contrary, the majority (60 out of 66, 90.9%) of those critical risk DEGs were hypoxia-related DEGs. These findings suggesting that immune status could play a favorable role in gastric cancer while hypoxia status might make adverse impacts on prognosis of gastric cancer patients. Further, Gene Ontology enrichment analyses found that the protective DEGs were related to activation of immune cells, migration of immune cells and release of inflammatory factors (Fig. 2e), while the risk DEGs could take part in cytoskeleton, cell junction and pathway involved in epithelial-mesenchymal transformation such as Wnt pathway (Fig. 2f).

**Fig. 3:**
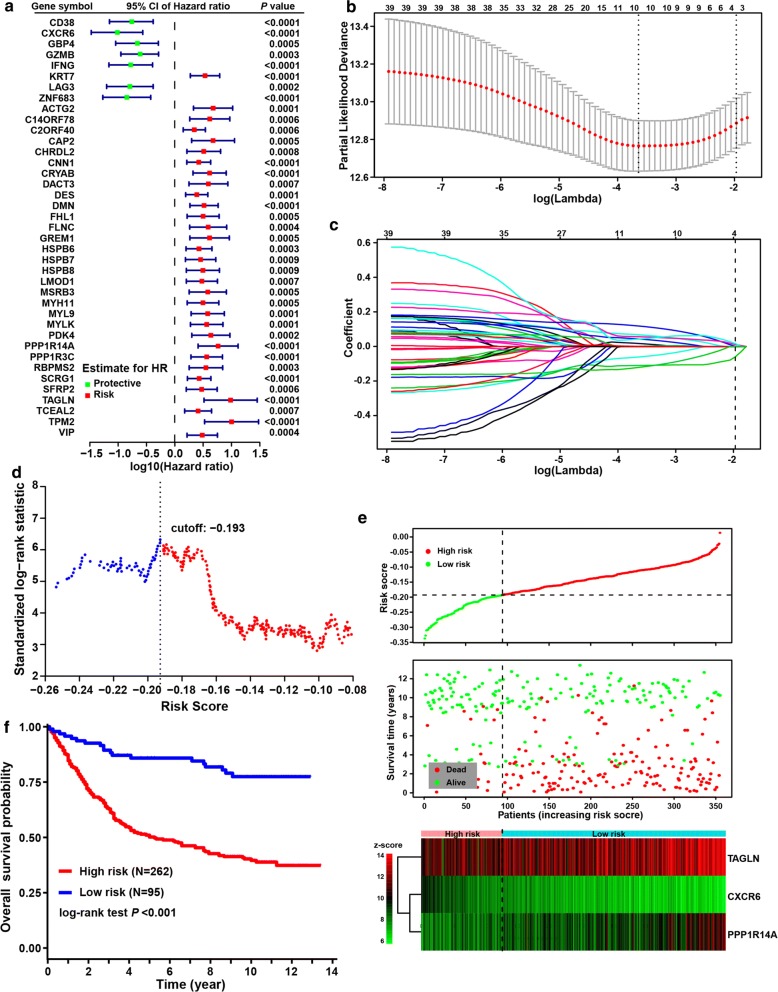
Hypoxia-immune-based gene signature and prognosis classifier. **a** Forest plot of hazard ratios for 39 hypoxia-immune-related prognostic DEGs. **b** Three-fold cross-validation for tuning parameter selection in the LASSO model. The partial likelihood deviance is plotted against log (λ), where λ is the tuning parameter. Partial likelihood deviance values are shown, with error bars representing SE. The dotted vertical lines are drawn at the optimal values by minimum criteria and 1-SE criteria. **c** LASSO coefficient profiles of the hypoxia-immune-related prognostic DEGs. The dotted line indicates the value chosen by 3-fold cross-validation. **d** Scatter plot shows the standardized log-rank statistic value for each corresponding cutoff of hypoxia-immune-based risk score. The optimal cutoff with the maximum standard log-rank statistic is marked with a vertical dashed line. **e** Distributions of risk score, survival status and expression profile of signature genes. **f** Kaplan–Meier plot of overall survival for patients in low-risk and high-risk groups by hypoxia-immune-based prognosis classifier in discovery cohort

To identified hypoxia-immune-related prognostic DEGs, univariate Cox regression analyses were further performed among all critical protective and risk DEGs. Among all of the 95 critical DEGs, 39 were identified with significantly effects on patient prognosis, including seven protective DEGs and 32 risk DEGs (Fig. 3a).

### Hypoxia-immune-based gene signature and prognosis classifier in gastric cancer

**Fig. 4:**
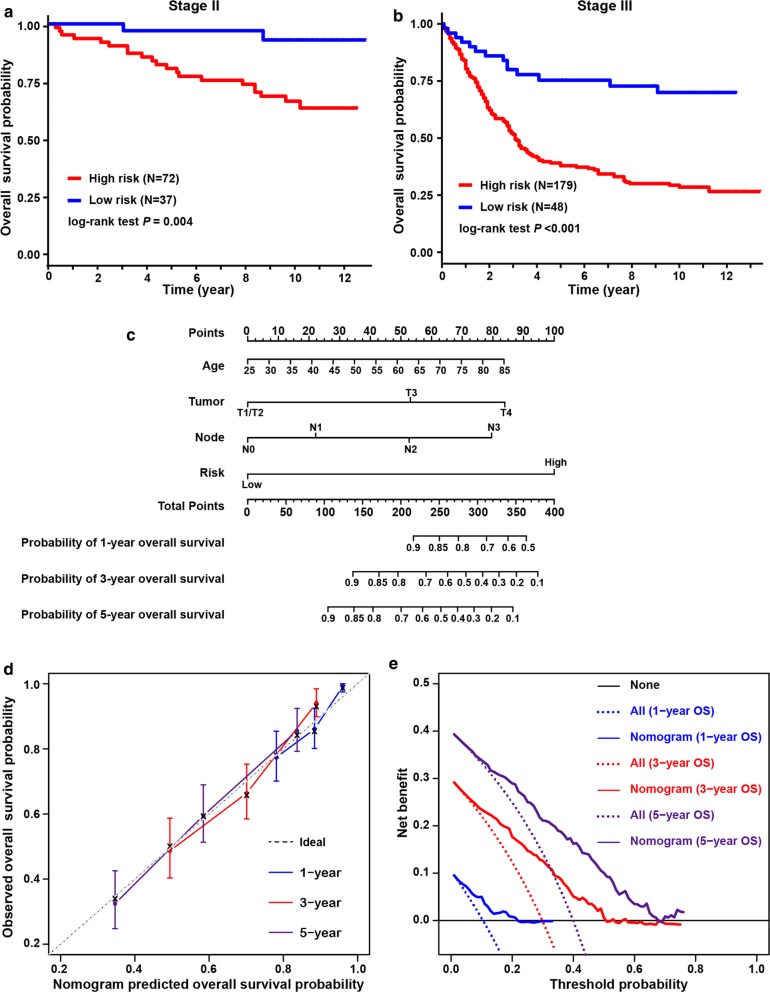
Validation of hypoxia-immune-based prognosis classifier in TNM subgroups and nomogram for predicting overall survival. **a**, **b** Kaplan–Meier plot of overall survival for patients in low-risk and high-risk groups by hypoxia-immune-based prognosis classifier in stage II and III subgroups in discovery cohort. **c** Nomogram developed by using discovery cohort to predict 1-, 3-, and 5-years overall survival probability. **d** Plot depicting the calibration of the nomogram in terms of the agreement between predicted and observed outcomes. Nomogram performance is shown by the plot relative to the dotted line, which represents perfect prediction. **e** Decision curve analysis shows the expected net benefits based on the nomogram prediction at different threshold probabilities. None: assume an event will occur in no patients (horizontal solid line); All: assume an event will occur in all patients (dash line)

The LASSO Cox regression model was employed to select the most useful prognostic gene signature from all hypoxia-immune-related prognostic DEGs within the discovery cohort (Fig. 3b) (see “Methods” for details). The optimal gene signature consisting of three prognostic DEGs (i.e., TAGLIN, CXCR6 and PPP1R14A) as well as the corresponding coefficients were identified (Fig. 3c). Among three signature genes, TAGLIN and PPP1R14A were risk DEGs and CXCR6 was protective. Expression levels of three signature DEGs and corresponding coefficients derived from the LASSO Cox regression model were used to calculate the individual-level risk score for each patient as following: risk score = − 0.059 × expression of CXCR6 + 0.011 × expression of PPP1R14A + 0.016 × expression of TAGLN. Based on this three-gene-based risk score, a prognosis classifier was developed to classify patients into high- and low-risk groups. The optimal risk score cutoff in this classifier was identified by the method of maximally selected rank statistics (Fig. 3d). The risk curve and signature DEGs expression pattern are plotted in Fig. 3e. Survival comparison showed that patients in the low-risk group yielded significant better survivals than those in high-risk group (log-rank test *p* < 0.001) (Fig. 3f). The prognosis classifier was further validated within two patient subgroups with stage II and stage III disease in the discovery cohort, respectively (Fig. 4a, b). Similarly, in both the stage II and stage III subgroups, patients were consistently stratified into the low-risk group with better survivals and the high-risk group yielding worse outcomes (log-rank test, *p* = 0.004 in stage II and *p* < 0.001 in stage III, respectively).

### Hypoxia-immune-based prognosis classifier and clinicopathologic characteristics in gastric cancer

The clinicopathologic characteristics of patients in the low- and high-risk groups are listed in Table 1. Gender, and age were comparable between the two risk groups. The high-risk group included more patients with advanced-stage tumors, and more patients in the low-risk group were with early-stage tumors (Chi square test, *p* = 0.004). Consistently with the abovementioned findings, the high-risk group tended to include more patients with high hypoxia status (χ^2^ test, *p* < 0.001) and low immune status (χ^2^ test, *p* < 0.001). Univariate analyses in the discovery cohort revealed that hypoxia and immune status, as well as the prognosis classifier were significant factors associated with survival (log-rank test, *p* < 0.001, *p* = 0.005 and *p* < 0.001, respectively). Multivariate analysis also identified the prognosis classifier as an independent prognostic factor (adjusted *p* < 0.001), which was similar to and independent of tumor stage (adjusted *p* < 0.001) (Table 3). This indicated the potential of integration of hypoxia and immune status for prognosis stratification in gastric cancer. Further, by integrating the hypoxia-immune-based prognosis classifier and well-known prognostic factors, a nomogram was constructed by using the discovery cohort for overall survival prediction (Fig. 4c). The C-index for nomogram was 0.782 (95% CI 0.703–0.861), significantly higher than that of TNM stage (0.665, 95% CI 0.634–0.696; *p* = 0.039), indicating better discrimination for the nomogram. The calibration curve showed well performance for the nomogram, compared with an ideal model (Fig. 4d). The clinical usefulness of the nomogram was quantified by the decision curve; the nomogram provided better net benefits than the alternative options, regarding 1-, 3-, and 5-years overall survival probability (Fig. 4e).

### Validation of hypoxia-immune-based prognosis classifier in three independent cohorts

**Fig. 5:**
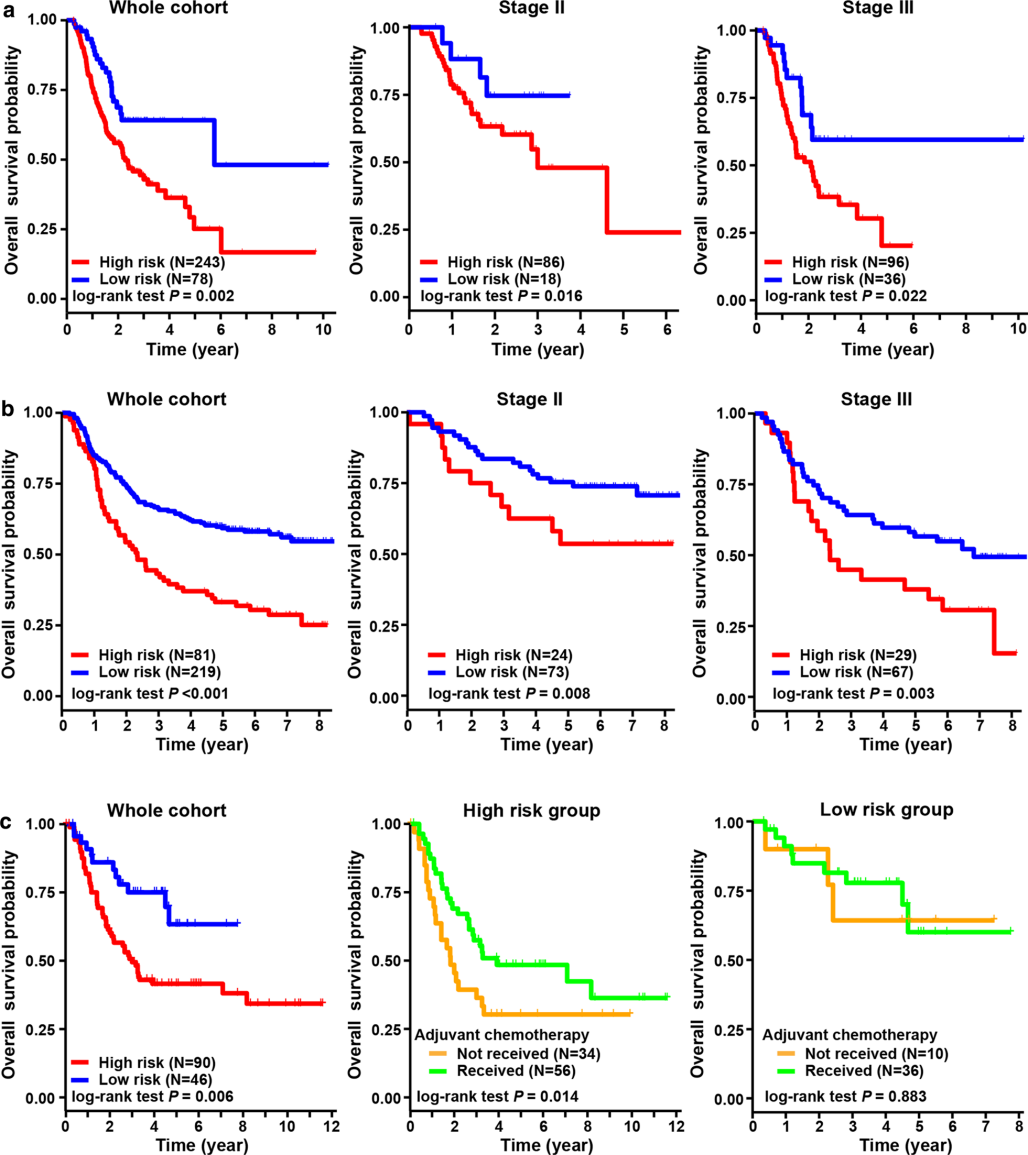
Validation of hypoxia-immune-based prognosis classifier in three independent cohorts regarding overall survival. **a** Kaplan–Meier plot of overall survival by risk groups for patients in the TCGA cohort and subgroups according to TNM staging. **b** Kaplan–Meier plot of overall survival by risk groups for patients in the ACRG cohort and subgroups according to TNM staging. **c** Kaplan–Meier plot of overall survival by risk groups for patients in the CHEM cohort. And overall survival comparison among patients received chemotherapy or not in the low- and high-risk groups

**Fig. 6:**
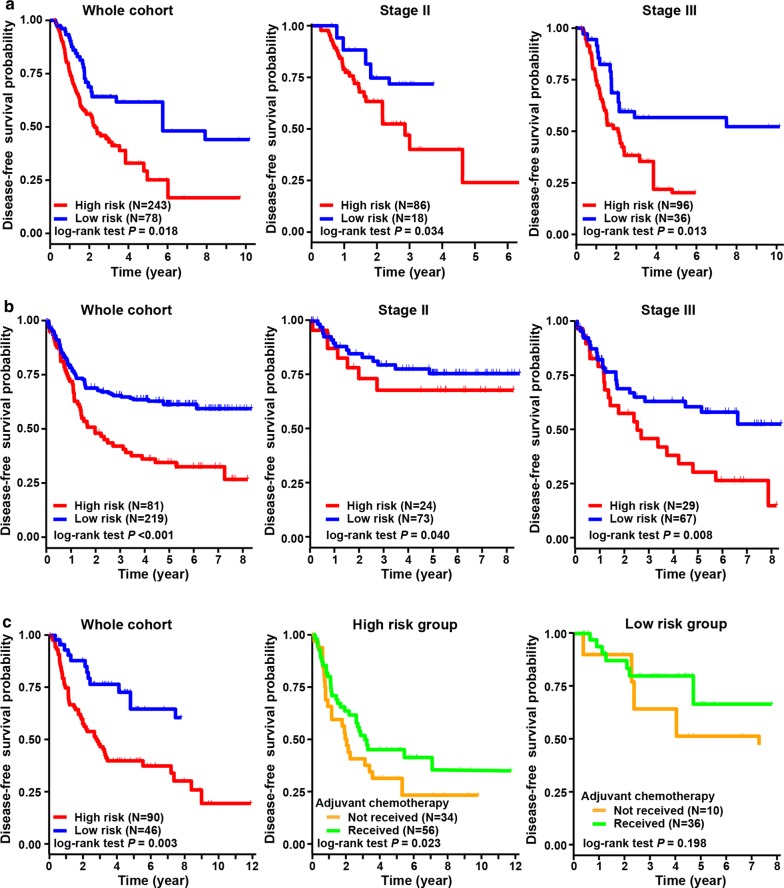
Validation of hypoxia-immune-based prognosis classifier in three independent cohorts regarding disease-free survival. **a** Kaplan–Meier plot of disease-free survival by risk groups for patients in the TCGA cohort and subgroups according to TNM staging. **b** Kaplan–Meier plot of disease-free survival by risk groups for patients in the ACRG cohort and subgroups according to TNM staging. **c** Kaplan–Meier plot of disease-free survival by risk groups for patients in the CHEM cohort. And disease-free survival comparison among patients received chemotherapy or not in the low- and high-risk groups

The hypoxia-immune-based prognosis classifier was further validated by three independent cohorts, including the TCGA, ACRG and CHEM cohorts (see Methods for details). Patient clinicopathologic characteristics are listed in Table 2. By employing the prognosis classifier, being similar with the findings in the discovery cohort, three validation cohorts were consistently stratified into the high-risk group with worse survivals and the low-risk group with better outcomes (log-rank test, *p* = 0.002 in TCGA cohort, *p* < 0.001 in ACRG cohort, and *p* = 0.006 in CHEM cohort, respectively) (Fig. 5). Validations were also conducted in subgroups of patients with stage II and III diseases in the TCGA and ACRG cohorts; similar results were also obtained. Interestingly, in the CHEM cohort, we found that patients in the high-risk group yielded survival benefits from adjuvant chemotherapy (log-rank test, *p* = 0.014); however, there were no significant survival benefits of chemotherapy for patients in the low-risk group (log-rank test, *p* = 0.883) (Fig. 5c). The efficacy of the classifier was further evaluated using another outcome of disease-free survival; and the results were similar with that of overall survival (Fig. 6). Consistently, multivariate analyses revealed that the prognosis classifier was an independent prognostic factor in three validation cohorts (adjusted *p* < 0.001 in TCGA cohort, adjusted *p* < 0.001 in ACRG cohort and adjusted *p* = 0.001 in CHEM cohort, respectively) (Table 3).

**Table 2:** Baseline characteristics of patients in three independent validation cohorts

Characteristics	Whole cohort	Low risk	High risk	*p*
TCGA cohort	(n = 321)	(n = 78)	(n = 243)	
Gender				0.772
Male	204 (63.55)	48 (61.54)	156 (64.20)	
Female	117 (36.45)	30 (38.46)	87 (35.80)	
Age				0.995
< 65 years	146 (45.48)	36 (46.15)	110 (45.27)	
≥ 65 years	175 (54.52)	42 (53.85)	133 (54.73)	
TNM stage^a^				0.253
I	44 (13.71)	13 (16.67)	31 (12.76)	
II	104 (32.40)	18 (23.08)	86 (35.39)	
III	132 (41.12)	36 (46.15)	96 (39.51)	
IV	29 (9.03)	7 (8.97)	22 (9.05)	
WHO classification				0.085
Intestinal/tubular	125 (38.94)	34 (43.59)	91 (37.45)	
Diffuse	57 (17.76)	15 (19.23)	42 (17.28)	
Mucinous/signet ring cell	26 (8.10)	1 (1.28)	25 (10.29)	
Others	113 (35.20)	28 (35.90)	85 (34.98)	

ACRG cohort	(n = 300)	(n = 219)	(n = 81)	
Gender				0.950
Male	199 (66.33)	146 (66.67)	53 (65.43)	
Female	101 (33.67)	73 (33.33)	28 (34.57)	
Age				0.999
< 65 years	161 (53.67)	118 (53.88)	43 (53.09)	
≥ 65 years	139 (46.33)	101 (46.12)	38 (46.91)	
TNM stage				0.097
I	30 (10.00)	27 (12.32)	3 (3.70)	
II	97 (32.33)	73 (33.33)	24 (29.63)	
III	96 (32.00)	67 (30.59)	29 (35.80)	
IV	77 (25.67)	52 (23.74)	25 (30.86)	
Lauren classification				0.141
Diffuse	135 (45.00)	91 (41.55)	44 (54.32)	
Intestinal	146 (48.67)	113 (51.60)	33 (40.74)	
Mixed	19 (6.33)	15 (6.84)	4 (4.93)	

CHEM cohort	(n = 136)	(n = 46)	(n = 90)	
Gender				0.709
Male	93 (68.38)	30 (65.22)	63 (70.00)	
Female	43 (31.62)	16 (34.78)	27 (30.00)	
Age				0.726
< 65 years	87 (63.97)	28 (60.87)	59 (65.56)	
≥ 65 years	49 (36.03)	18 (39.13)	31 (34.44)	
TNM stage				0.496
II	36 (26.47)	15 (32.61)	21 (23.33)	
III	63 (46.32)	19 (41.30)	44 (48.89)	
IV	37 (27.21)	12 (26.09)	25 (27.78)	
Lauren classification^a^				0.075
Diffuse	30 (22.06)	15 (32.61)	15 (16.67)	
Intestinal	95 (69.85)	27 (58.70)	68 (75.56)	
Mixed	7 (5.15)	3 (6.52)	4 (4.44)	

^a^Twelve patients with unavailable stage information in the TCGA cohort and four patients with unavailable Lauren classification information in the CHEM cohort were excluded from the comparison

**Table 3:** Multivariate Cox regression analyses of risk factors for overall survival

	Adjusted Hazard ratio^a^	95% confidence interval	Adjusted *p*
Discovery cohort			
Risk group (High vs low)	3.24	2.03–5.18	< 0.001
Tumor stage			< 0.001
II vs I	1.35	0.48–3.81	0.574
III vs I	3.37	1.24–9.17	0.017
TCGA cohort			
Risk group (High vs low)	2.91	1.76–4.80	< 0.001
Tumor stage			< 0.001
II vs I	1.91	0.93–3.92	0.076
III vs I	3.04	1.54–6.01	0.001
VI vs I	6.92	3.07–15.59	< 0.001
ACRG cohort			
Risk group (High vs low)	1.99	1.42–2.78	< 0.001
Tumor stage			< 0.001
II vs I	1.51	0.58–3.93	0.398
III vs I	3.01	1.18–7.68	0.021
VI vs I	8.83	3.50–22.28	< 0.001
CHEM cohort			
Risk group (High vs low)	3.01	1.56–5.83	0.001
Tumor stage			0.012
III vs II	2.20	1.04–4.64	0.039
IV vs II	3.35	1.50–7.48	0.003

^a^Gender and age were adjusted for modelling using the discovery cohort; and gender, age and histology classification were adjusted when using TCGA, ACRG and CHEM cohorts

## Discussion

Considering the widely varying prognostic outcomes of gastric cancer, it is of great importance to establish a robust classifier to stratify patients with different risks and prognoses, which is critical to maximize the benefits brought by the personalized treatment and timely follow-up. In this study, the comprehensive mining of the transcriptional profiles and microenvironment characteristics was aimed to construct a tool to help address this important clinical issue. We found that both the hypoxia and immune status of the tumor microenvironment was associated with gastric cancer patient survival. Moreover, the inverse effects of hypoxia and immune status were significantly associated with prognosis, even after stratifying patients by clinicopathologic risk factors. Finally, a hypoxia-immune-based three-gene signature was developed as a prognosis classifier, with promising performance in risk stratification among the discovery cohort and three independent cohorts. These findings represent a new insight to improve discussions on patient prognostication and stratification through considering the microenvironment characteristics and transcriptomics.

The immune and hypoxia microenvironment plays a critical role in the tumorigenesis and progression of gastric cancer [10, 11, 13–15, 25]. On one hand, the immune incapability in the tumor microenvironment has been reported as an essential mechanism for solid cancers to evade from host immunity [13, 15, 25–27]. It was found that higher expression of immune-related gene predicts better prognosis in both EBV-positive and EBV-negative gastric cancer patients [25]. Also, the estimation of immune cells in tumor tissues could improve the accuracy of TNM staging system for prognostication in gastric cancer [28]. On the other hand, the hypoxic microenvironment promotes tumor malignancy by activating angiogenesis and increases cell migration and expansion toward cancer stem cell phenotype by altering cell skeleton and extracellular matrix [10–12]. These findings were similar with the results in current study. We found that the protective DEGs, mainly containing the immune-related DEGs, could take part in the activation of immune cells, migration of immune cells and release of inflammatory factors and those risk DEGs, of which the majority was hypoxia-related DEGs, were associated with cytoskeleton, cell junction and pathway involved in epithelial-mesenchymal transformation such as Wnt pathway. More importantly, it has been reported that hypoxia incapacitated immune effector cells [17] and enhanced the activity of immunosuppressive cells [16], and immune escape [29] and tumor cell adaptations to hypoxia [30] could promote and perpetuate immunosuppression. In current analysis, we found that the hypoxia and immune status made inverse effects on patient prognosis in gastric cancer; higher hypoxia status was associated with poor prognosis while higher immune status could indicate better outcomes; and the impact of the inverse interaction on survival also observed after combining hypoxia and immune status. Thus, the hypoxia and immune status accompanied with their interaction in tumor microenvironment and its linking to gastric cancer progression could provide improved discussion with gastric cancer regarding prognosis.

As the hypoxia and immune activity in tumor microenvironment is complicated, there is no public biomarker using mRNA expression pattern to estimate their status [31, 32]. Indeed, as tumors develop regions of hypoxia, tumor can also react favorably to hypoxic conditions as well as recovery of tumor blood and nutrient supply to some extent [32, 33]. Thus, it is not powerful (and likely to omit important biology process information) to determine the hypoxia status by a single biomarker [10, 11, 34]. The machine learning algorithm *t*-SNE provides an elegant and robust dimensionality reduction approach, which has been applied to explore potential subtypes in prostate cancer [35] and breast cancer [36]. In the present study, the nonlinear cluster method of *t*-SNE identified distinct patterns of hypoxic tumor microenvironment based on a set of two hundred hypoxia hallmark genes; further, expression changes of HIF-1 targeting genes were analyzed to explore their association with the hypoxia process. When it came to immune status, the ESTIMATE algorithm was used to impute immune scores to predict the level of infiltrating immune cells based on 141 specific gene signatures of immune cells. It was a newly developed algorithm that takes advantage of the unique properties of the transcriptional profiles of cancer tissues to infer tumor cellularity as well as the different infiltrating normal cells [21]. Subsequent works have applied the ESTIMATE algorithm to prostate cancer [37], breast cancer [38], and colon cancer [39], showing the effectiveness of such big-data based algorithms, although combination of immune characteristics with hypoxia status has not been investigated in detail.

Important roles of the signature genes identified in this study have been previously reported in multiple types of cancers. TAGLN encodes a shape change and transformation sensitive actin-binding protein. Overexpression of TAGLN was associated with cell invasion, which in turn contributed to promoting cancer metastasis [40]. Notably, the expression of TAGLN is significantly induced by hypoxia in lung adenocarcinoma [41]. Another risk gene PPP1R14A, has been reported to drive Ras pathway and tumorigenesis via inactivation of the tumor suppressor merlin [42]. These results were consistent with the results in this study that overexpression of TAGLN and PPP1R14A could be unfavorable factors for patient’s outcomes. The protective gene CXCR6 is known as a chemokine receptor, which is selectively expressed in NK cells, T cells, and plasma cells. It is responsible for the chemotactic migration of immune cells to cancer tissues, which has the potential to kill cancer cells [43, 44]. In our study, CXCR6 was identified as an immune-related, favorable gene for prognosis in gastric cancer. However, the abovementioned three signature genes were seldom studied in the context of combination of immune and hypoxia. Thus, signature genes identified in this study could provide underlying targets for experimental design in the laboratory to elucidate molecular mechanisms in gastric cancer.

Many potential targets, even with their detailed mechanism of action, have been revealed to play critical roles in tumor development and progression. However, it remains challenging for clinicians and researchers to translate these efforts and findings from laboratory into clinical settings. Integration of molecular and genetic characteristics and clinicopathologic factors provides a new insight for this issue regarding precision prognostication and individualized treatment. In current study, it was worth mentioning that patients in the low-risk group seemed not benefit from adjuvant chemotherapy. This provides a hint that hypoxia and immune status could serve as underlying markers for selecting sensitive patients to chemotherapy. Ongoing efforts on characterizing properties of tumor cell and its microenvironment are making intrinsic and extrinsic variations more and more clear; meanwhile, emerging techniques of sequencing make it possible for individualized risk stratification and treatment at molecular level in clinical application. Thus, the findings in current study, which links the genetic profiles and microenvironment characteristics to patient prognosis, could potentially provide translational value for clinical management of patients with gastric cancer.

Several limitations exist in this study. First, although several independent external validations were performed in this study, it was difficult to cover all variations among patients from different geographical regions when tissues and information were retrospectively collected in publicly available databases. Second, considering that the microenvironment characteristics might be distinct in different tumor regions, such as tumor core and invasive margin. Samples used for analyses were all collected from the core of tumor, and it is impossible to evaluate the immune and hypoxia status in different tumor regions. Thus, findings in this study are waiting for further validation by well-designed, prospective, multicenter studies.

## Conclusions

In conclusion, the hypoxia and immune status in tumor microenvironment is associated with the prognosis of gastric cancer patients. And the hypoxia-immune-based gene signature yielded promising ability for risk stratification and can providing additional value beyond the current TNM staging system. It might serve as a prognostic classifier for clinical decision-making regarding individualized prognostication and treatment, and follow-up scheduling.

## Data Availability

All data generated or analyzed during this study are included in this published article.

## Abbreviations

TNM: Tumor-node-metastasis
GEO: Gene expression omnibus database
TCGA: The Cancer Genome Atlas
ACRG: Asian Cancer Research Group
FPKM: Fragments per kilobase of exon per million reads mapped
*t*-SNE: *t*-Distributed stochastic neighbor embedding
MSigDB: Molecular signatures database
GSEA: Gene set enrichment analysis
DEGs: Differentially expressed genes
FDR: False discovery rate
ESTIMATE: Estimation of STromal and Immune cells in MAlignant Tumor tissues using Expression data
LASSO: The least absolute shrinkage and selection operator

## References

[1] Siegel RL, Miller KD, Jemal A (2019). Cancer statistics, 2019. Cancer J Clin.

[2] Ajani JA, D’Amico TA, Almhanna K, Bentrem DJ, Chao J, Das P (2016). Gastric cancer, Version 32016, NCCN Clinical Practice Guidelines In Oncology. JNCCN..

[3] Shah MA, Ajani JA (2010). Gastric cancer–an enigmatic and heterogeneous disease. JAMA.

[4] Noh SH, Park SR, Yang HK, Chung HC, Chung IJ, Kim SW (2014). Adjuvant capecitabine plus oxaliplatin for gastric cancer after D2 gastrectomy (CLASSIC): 5-year follow-up of an open-label, randomised phase 3 trial. Lancet Oncol..

[5] Hanahan D, Coussens LM (2012). Accessories to the crime: functions of cells recruited to the tumor microenvironment. Cancer Cell.

[6] Hanahan D, Weinberg RA (2011). Hallmarks of cancer: the next generation. Cell.

[7] Becht E, Giraldo NA, Germain C, de Reynies A, Laurent-Puig P, Zucman-Rossi J (2016). Immune contexture, immunoscore, and malignant cell molecular subgroups for prognostic and theranostic classifications of cancers. Adv Immunol.

[8] Vaupel P, Kelleher DK, Thews O (1998). Modulation of tumor oxygenation. Int J Radiat Oncol Biol Phys.

[9] Harris AL (2002). Hypoxia–a key regulatory factor in tumour growth. Nat Rev Cancer.

[10] Shida M, Kitajima Y, Nakamura J, Yanagihara K, Baba K, Wakiyama K (2016). Impaired mitophagy activates mtROS/HIF-1alpha interplay and increases cancer aggressiveness in gastric cancer cells under hypoxia. Int J Oncol.

[11] Nam SY, Ko YS, Jung J, Yoon J, Kim YH, Choi YJ (2011). A hypoxia-dependent upregulation of hypoxia-inducible factor-1 by nuclear factor-kappaB promotes gastric tumour growth and angiogenesis. Br J Cancer.

[12] Gilkes DM, Semenza GL, Wirtz D (2014). Hypoxia and the extracellular matrix: drivers of tumour metastasis. Nat Rev Cancer.

[13] Wei M, Shen D, Mulmi Shrestha S, Liu J, Zhang J, Yin Y (2018). The progress of T cell immunity related to prognosis in gastric cancer. Biomed Res Int.

[14] Thompson ED, Zahurak M, Murphy A, Cornish T, Cuka N, Abdelfatah E (2017). Patterns of PD-L1 expression and CD8 T cell infiltration in gastric adenocarcinomas and associated immune stroma. Gut.

[15] Lazar DC, Avram MF, Romosan I, Cornianu M, Taban S, Goldis A (2018). Prognostic significance of tumor immune microenvironment and immunotherapy: novel insights and future perspectives in gastric cancer. World J Gastroenterol.

[16] Deng B, Zhu JM, Wang Y, Liu TT, Ding YB, Xiao WM (2013). Intratumor hypoxia promotes immune tolerance by inducing regulatory T cells via TGF-beta1 in gastric cancer. PLoS ONE.

[17] Noman MZ, Desantis G, Janji B, Hasmim M, Karray S, Dessen P (2014). PD-L1 is a novel direct target of HIF-1alpha, and its blockade under hypoxia enhanced MDSC-mediated T cell activation. J Exp Med.

[18] Raie AL (2003). Exploration, normalization, and summaries of high density oligonucleotide array probe level data. Biostatistics..

[19] Krijthe J. Rtsne: T-distributed Stochastic Neighbor Embedding using Barnes-Hut implementation. 2016. https://cran.r-project.org/web/packages/Rtsne.

[20] Ritchie ME, Phipson B, Wu D, Hu Y, Law CW, Shi W (2015). limma powers differential expression analyses for RNA-sequencing and microarray studies. Nucleic Acids Res.

[21] Yoshihara K, Shahmoradgoli M, Martinez E, Vegesna R, Kim H, Torres-Garcia W (2013). Inferring tumour purity and stromal and immune cell admixture from expression data. Nat Commun.

[22] Hothorn T, Zeileis A (2008). Generalized maximally selected statistics. Biometrics.

[23] Tibshirani R (1997). The lasso method for variable selection in the Cox model. Stat Med.

[24] Zhang JX, Song W, Chen ZH, Wei JH, Liao YJ, Lei J (2013). Prognostic and predictive value of a microRNA signature in stage II colon cancer: a microRNA expression analysis. Lancet Oncol.

[25] Sundar R, Qamra A, Tan ALK, Zhang S, Ng CCY, Teh BT (2018). Transcriptional analysis of immune genes in Epstein–Barr virus-associated gastric cancer and association with clinical outcomes. Gastric Cancer.

[26] Hao D, Liu J, Chen M, Li J, Wang L, Li X (2018). Immunogenomic analyses of advanced serous ovarian cancer reveal immune score is a strong prognostic factor and an indicator of chemosensitivity. Clin Cancer Res.

[27] Gao Y, Rae W, Ramakrishnan KA, Barcenas-Morales G, Doffinger R, Eren E (2016). Mucosal-associated invariant T (MAIT) cells are impaired in Th17 associated primary and secondary immunodeficiencies. PLoS ONE.

[28] Wen T, Wang Z, Li Y, Li Z, Che X, Fan Y (2017). A four-factor immunoscore system that predicts clinical outcome for stage II/III gastric cancer. Cancer Immunol Res.

[29] Zhang H, Lu H, Xiang L, Bullen JW, Zhang C, Samanta D (2015). HIF-1 regulates CD47 expression in breast cancer cells to promote evasion of phagocytosis and maintenance of cancer stem cells. Proc Natl Acad Sci USA.

[30] Siska PJ, Rathmell JC (2015). T cell metabolic fitness in antitumor immunity. Trends Immunol.

[31] Staudacher JJ, Naarmann-de Vries IS, Ujvari SJ, Klinger B, Kasim M, Benko E (2015). Hypoxia-induced gene expression results from selective mRNA partitioning to the endoplasmic reticulum. Nucleic Acids Res.

[32] Pugh CW (2016). Modulation of the hypoxic response. Adv Exp Med Biol.

[33] Fukumura D, Jain RK (2007). Tumor microvasculature and microenvironment: targets for anti-angiogenesis and normalization. Microvasc Res.

[34] Liu L, Zhao X, Zou H, Bai R, Yang K, Tian Z (2016). Hypoxia promotes gastric cancer malignancy partly through the HIF-1alpha dependent transcriptional activation of the long non-coding RNA GAPLINC. Front Physiol.

[35] Ahmed M, Lai TH, Zada S, Hwang JS, Pham TM, Yun M (2018). Functional linkage of RKIP to the epithelial to mesenchymal transition and autophagy during the development of prostate cancer. Cancers..

[36] Guo L, Chen G, Zhang W, Zhou L, Xiao T, Di X (2019). A high-risk luminal A dominant breast cancer subtype with increased mobility. Breast Cancer Res Treat.

[37] Shah N, Wang P, Wongvipat J, Karthaus WR, Abida W, Armenia J (2017). Regulation of the glucocorticoid receptor via a BET-dependent enhancer drives antiandrogen resistance in prostate cancer. eLife..

[38] Priedigkeit N, Watters RJ, Lucas PC, Basudan A, Bhargava R, Horne W (2017). Exome-capture RNA sequencing of decade-old breast cancers and matched decalcified bone metastases. JCI insight..

[39] Alonso MH, Ausso S, Lopez-Doriga A, Cordero D, Guino E, Sole X (2017). Comprehensive analysis of copy number aberrations in microsatellite stable colon cancer in view of stromal component. Br J Cancer.

[40] Yu B, Chen X, Li J, Qu Y, Su L, Peng Y (2013). Stromal fibroblasts in the microenvironment of gastric carcinomas promote tumor metastasis via upregulating TAGLN expression. BMC Cell Biol..

[41] Wu X, Dong L, Zhang R, Ying K, Shen H (2014). Transgelin overexpression in lung adenocarcinoma is associated with tumor progression. Int J Mol Med.

[42] Lars Björn Riecken AZ, Ulrike W, Sabine R (2016). CPI-17 drives oncogenic Ras signaling in human melanomas via Ezrin–Radixin–Moesin family proteins. Oncotarget..

[43] Mossanen JC, Kohlhepp M, Wehr A, Krenkel O, Liepelt A, Roeth AA (2019). CXCR6 inhibits hepatocarcinogenesis by promoting natural killer T- and CD4(+) T-cell-dependent control of senescence. Gastroenterology.

[44] Won EJ (2016). Clinical relevance of circulating mucosal-associated invariant T cell levels and their anti-cancer activity in patients with mucosal-associated cancer. Oncotarget.

